# Screen Position Preference Offers a New Direction for Action Observation Research: Preliminary Findings Using TMS

**DOI:** 10.3389/fnhum.2018.00026

**Published:** 2018-02-01

**Authors:** Martin Riach, David J. Wright, Zoë C. Franklin, Paul S. Holmes

**Affiliations:** Research Centre for Musculoskeletal Science and Sports Medicine, Manchester Metropolitan University, Manchester, United Kingdom

**Keywords:** action observation, transcranial magnetic stimulation, motor-evoked potentials, spatial reference frames, screen position preference

## Abstract

Action observation has been suggested to be an effective adjunct to physical practice in motor (re)learning settings. However, optimal viewing conditions for interventions are yet to be established. Single-pulse transcranial magnetic stimulation (TMS) was used to investigate the effect of two different screen positions and participants’ screen position viewing preference on the amplitude of motor evoked potentials (MEPs) during observation of a ball pinch action. Twenty-four participants observed four blocked conditions that contained either a dynamic index finger-thumb ball pinch or a static hand holding a ball in a similar position on a horizontally or vertically positioned screen. TMS was delivered to the hand representation of the left primary motor cortex and MEPs were recorded from the first dorsal interosseous muscle of the right hand. Initial analysis of the normalized MEP amplitude data showed no significant differences between conditions. In a follow-up procedure, participants engaged in individual semi-structured interviews and completed a questionnaire designed to assess viewing affect and screen position viewing preference. The MEP data were subsequently split by screen position preference and re-analyzed using a 2 × 2 repeated measures ANOVA. Main effects indicated that participants who preferred the horizontal screen position (*n* = 16) demonstrated significantly greater MEP amplitudes during observation of the ball-pinch action compared to the static hand condition irrespective of screen position, and during the horizontal compared to the vertical screen position irrespective of video type. These results suggest that ensuring anatomical and perceptual congruency with the physical task, alongside consideration of participants’ screen position viewing preferences, may be an important part of optimizing action observation interventions.

## Introduction

Action observation interventions have been shown to contribute effectively to improvements in movement function in motor (re)learning settings (for a review see Buccino, 2014). An observation-execution matching mechanism has been argued to map the observed action onto the observer’s own motor representations, with the implicit assumption being that a more active extended motor system during action observation is more effective for motor (re)learning (for a recent review, see Eaves et al., 2016). In response, researchers are exploring the effects of different movement simulation methods on activity in the motor system in an attempt to optimize the design and delivery of action observation interventions (Holmes and Wright, 2017).

Transcranial magnetic stimulation (TMS) has been used extensively to explore corticospinal activity in the motor system during action observation, with the amplitude of the motor evoked potential (MEP) response providing a marker of corticospinal excitability (Ruffino et al., 2017). Experiments using TMS have shown that the MEP amplitude increases during the observation of hand and arm movements when compared to various control conditions (Fadiga et al., 1995), and has been replicated across a variety of movement tasks (for reviews, see Loporto et al., 2011; Naish et al., 2014).

One variable that has been suggested to modulate corticospinal excitability during action observation is visual perspective. Several studies have reported that corticospinal excitability is facilitated to a greater extent when participants observe actions from a first person visual perspective compared to third person visual perspectives (e.g., Maeda et al., 2002; Alaerts et al., 2009), even when both visual perspectives show movements made by another individual. The first person visual perspective aims to create an action observation condition that mimics a “self-based” movement in contrast to the third person visual perspective which is more clearly “other-based”. Importantly, increasing the perception of observing a self-based action by combining a first person visual perspective with an egocentric spatial reference frame (e.g., by matching the observed action with the observer’s spatial positioning) has been shown to evoke stronger neural activity in the observer’s extended motor system (Vogeley and Fink, 2003) and is consistent with Jeannerod’s Simulation Theory (Jeannerod, 2001). Results from visual perspective-based research suggest that action observation interventions filmed from a first person visual perspective may be more effective at promoting functional neural change than interventions filmed from third person visual perspectives (for a recent review, see Ruffino et al., 2017).

Despite the evidence supporting the use of a combined first person visual perspective and egocentric reference frame for action observation, a vertical screen position has been the most frequently employed position for presenting videos in action observation experiments using TMS, although there are some exceptions (e.g., Kaneko et al., 2007; Wright et al., 2016). Practically, the use of a vertical screen position is understandable as television and computer screens are generally orientated in this plane and we view others’ actions from a similar reference frame. However, when using vertically positioned screens to present first person visual perspective self-agency actions, the observed movement is viewed from an allocentric reference frame that is incongruent to the observer’s viewing position. We propose, therefore, that for observation interventions attempting to create an egocentric, first person visual perspective, a vertical screen presents a less embodied, more “detached” action relative to the observer’s own body. In the case of viewing a forearm and hand aiming to mimic the viewer’s own limb and hand, the presented action becomes an upward rotation (90°) in the sagittal plane away from the more anatomically-accurate transverse plane in which self-executed hand and arm actions are normally viewed and executed. The manipulation retains visual congruence with the task but the vertical reference frame could make the movement seem biomechanically-impossible due to the limb rotation and displacement of the observed action from the observer’s body. Consequently, this may alter the motor response during action observation; an argument which has been supported by a number of research groups (e.g., Romani et al., 2005; Borroni et al., 2011). In addition, and in a similar way to the visual perspective-agency confound, the different reference frame in vertical screen conditions presents an action with reduced visual cues for self-agency and a sense of ownership (Jeannerod and Pacherie, 2004) despite the action being presented in a first person visual perspective and aiming to represent the viewer’s limb. Therefore, in order to represent the action with the more congruent egocentric reference frame, and thereby retain a greater perception of self-agency and ownership with the presented action, the observer may make mental adjustments to the observed action, what Filimon (2015) terms “ego-relative remapping” (p. 2). Specifically, we propose that the observer may have to use concurrent coordinative motor imagery (Vogt et al., 2013) during the action observation condition to rotate the observed action and reinstate an anatomically-accurate egocentric reference frame that is more congruent with the physical action characteristics.

Given these concerns, it would seem paradoxical that research continues to use vertical screen presentations for action observation where the aim is to provide a first person visual perspective with a promotion of ownership and self-agency mechanisms to access the motor representation optimally (Jeannerod, 2001). We argue here that delivery of action observation through vertically orientated screens mimics an other-based movement, even if the action is filmed from a first person visual perspective; the incongruence between the observer’s own hand and the observed action inadvertently promotes less ownership and a greater sense of “other” agency. Consequently, we propose that a first person visual perspective video, observed on a horizontally angled screen located in the observer’s peripersonal space provides a more accurate egocentric reference frame for observation of hand and arm movements in research and applied interventions.

We have offered arguments for why screen position, based on differing reference frames, may influence corticospinal excitability during action observation. However, participants’ screen position viewing preferences based on the associated affect may also need consideration. In the imagery literature, preference for using a specific visual perspective (i.e., first person or third person) is an accepted methodological manipulation variable (Hall, 1997; Calmels et al., 2006) and, as such, participants’ visual perspective preference has become an important consideration when designing imagery interventions. Given that motor imagery and action observation share partial neural substrate and elicit some common activity in the motor system (Hétu et al., 2013), individuals may also have viewing preferences during action observation in a similar way to imagery perspective preference. For example, in one of the few studies to consider action observation viewing preferences, Ustinova et al. (2010), using a third person visual perspective video, manipulated the viewing angles of an avatar during a reaching movement. Following viewing, participants indicated a preference for observing the movement at greater angles (i.e., 45° or higher). If action observation viewing preference is evident within a third person visual perspective, preference may also exist for a first person visual perspective action observed from different reference frames and may reveal corticospinal excitability differences during different action observation conditions.

Taken together, there would seem to be a strong case to consider the corticospinal response of individuals viewing the same action on vertically and horizontally orientated screens, whilst also considering the influence of each participant’s affect on action observation viewing preference. The first aim of this experiment was to determine whether different screen positions modulate corticospinal excitability during observation of hand movements filmed from a first person visual perspective and aiming to present self-agency. The second aim was to establish whether corticospinal excitability was modulated when accounting for participants’ viewing preference for screen position. It was hypothesized that a first person visual perspective video viewed on a horizontal screen would facilitate corticospinal excitability to a greater extent than the same video observed on a vertical screen. Given the complexity of the inter-relationships, no directional hypotheses were made for viewing preference and corticospinal excitability.

## Materials and Methods

### Participants

Twenty-four individuals (16 male, 8 female) aged 19–37 years (mean age 23.96 ± 4.41 years) participated in the experiment. Eighteen participants were right-handed and six were left-handed, as measured by the Edinburgh Handedness Inventory (Oldfield, 1971). Previous research has successfully explored corticospinal excitability during action observation whilst including left-handed participants (e.g., Romani et al., 2005; Enticott et al., 2010; Wright et al., 2018), so handedness was not treated as an inclusion criteria. The TMS Adult Safety Screen (Keel et al., 2001) was used to ensure that no participants were predisposed to possible adverse effects of the stimulation. No participants were excluded based on these criteria and none reported discomfort or negative reactions during the experiment. The protocol was approved by the Manchester Metropolitan University local ethics committee. All subjects gave written informed consent in accordance with the Declaration of Helsinki (World Medical Association, 2013).

### Electromyography and Transcranial Magnetic Stimulation Protocol

#### Electromyography (EMG)

Electromyographic recordings were collected from the mid-point of the muscle belly of the first dorsal interosseous (FDI) of the right hand using a bipolar, single differential surface electrode (DE-2.1, Delsys Inc, Boston, MA, USA). The FDI muscle was chosen as it is actively involved in the execution of the observed action. A reference electrode was attached over the right ulnar process. Electrode sites were cleaned using alcohol wipes prior to attachment. The electromyography (EMG) signal was recorded using Spike2 version 6.18 software (Cambridge Electronic Design, Cambridge, UK) via a Micro 1401-3 analog-to-digital converter (Cambridge Electronic Design, Cambridge, UK), with a sampling rate of 2 kHz, bandwidth of 20 Hz to 450 kHz, 92 dB common mode rejection ratio and >10^15^ Ω input impedance.

#### Transcranial Magnetic Stimulation (TMS)

A figure-of-eight coil (two 70 mm diameter loops) was used to deliver the stimulation from a Magstim 200^2^ magnetic stimulator (Magstim Co., Whitland, Dyfed, UK), delivering monophasic pulses with a maximum field strength of 2.2 Tesla. The TMS procedure followed the published guidelines of Loporto et al. (2011). The coil was held in place over the hand representation of the left motor cortex with a mechanical arm (Manfrotto UK Limited, Ashby-de-la-Zouch, England) and was orientated for the induced current to flow in a posterior-anterior direction by positioning the coil at a 45° angle to the midline between nasion and inion landmarks of the skull. This coil orientation was used to achieve indirect trans-synaptic activation and optimal MEP amplitudes (Sakai et al., 1997; Opitz et al., 2013). The optimal scalp position (OSP) was found by stimulating the approximate area of the motor cortex for the FDI muscle of the right hand at 60% of the maximum stimulator output (Wright et al., 2014). The coil was then moved in 1 cm steps around this area until the site that produced MEPs of largest amplitude in the FDI muscle was found. This area was then marked on a tightly-fitting cap worn by the participants to ensure consistent coil placement throughout the experiment. After determining the OSP, the resting motor threshold (RMT) was determined by gradually adjusting the stimulation intensity until peak-to-peak MEP amplitudes of 50 μV or less were found in 5 out of 10 trials (Rossini et al., 2015). This stimulation intensity plus 1% maximum stimulator output was defined as the RMT (Rossini et al., 2015). The experimental stimulation intensity was then set at 110% RMT to reduce direct wave stimulation (Loporto et al., 2013). Experimental stimulation intensity ranged between 43% and 72% of the maximum stimulator output (mean intensity 52.63% ± 6.87).

### Procedure

Participants were seated in a dimly lit room with their elbows flexed at 90° and their hands pronated in a relaxed position. The participant’s right hand was positioned on a table directly in front of them under a black-painted wooden box, and their left hand relaxed on the table. A chinrest and headrest was used to limit head movements. Participants were asked to refrain from any voluntary movement during each condition and to attend fully to the stimuli presented on the screen. Blackout curtains were drawn alongside the screen and table setup to reduce any distracting visual stimuli.

**Figure 1:**
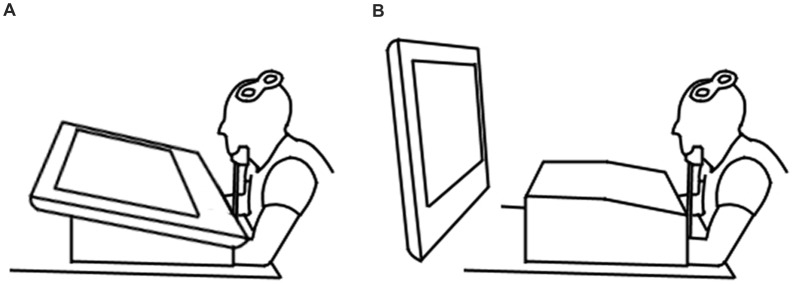
Experimental setup demonstrating the horizontal **(A)** and vertical **(B)** screen positions.

Each participant took part in four conditions. The two experimental conditions involved observation of an index finger-thumb pinch of a blue foam ball on either a horizontally positioned (15° to the table, distance of 45 cm, Figure 1A) or a vertically positioned (90° to the table, distance of 90 cm, Figure 1B) 32^″^ LCD screen (DGM Model LTV-3203H). The two control conditions required observation of a static hand holding the same ball between the index finger and thumb on either the horizontally or vertically positioned screen. Conditions were split into four blocks of 30 trials, with each block containing 15 action observation and 15 static control trials presented in a random order, resulting in a total of 30 trials per condition (Cuypers et al., 2014). Two blocks were presented for the horizontally positioned screen and two for the vertically positioned screen. The screen position presentation order was randomized.

The action observation and static hand videos showed a right hand and forearm filmed from a first person visual perspective and with the hand positioned to the right of the screen to give the visual appearance of the observed arm and hand being positioned similarly to the participant’s own limb. A Caucasian female arm/hand with no discernible features was used for the model in all videos to keep the observed movement consistent across conditions. A post-experiment manipulation check confirmed that the majority of participants perceived the observed hand to be of their own sex (71%). The action and static videos were of 10 s duration, with the action observation video containing four pinches at a rate of 0.4 Hz. Using a bespoke script run through the Spike2 software, one stimulation was delivered per trial at the point of maximal FDI flexion during the second or third ball pinch, and at the same time points during the static videos (either 3650 ms or 6150 ms after video onset). Two stimulation timings were used to reduce the predictability of the stimulation (Loporto et al., 2012). There were 64 stimulations for each time point. Participants were given a break of approximately 2 min between each block.

On completion of the TMS protocol, the lead author conducted a one-to-one deductive semi-structured interview with each participant to explore his/her experiences of each screen position. Questions targeted action observation experiences such as visual perspective, movement agency, movement kinesthesis and peripersonal space to analyze commonly recurring themes in action observation and motor imagery research (for a review of these themes, see Holmes and Calmels, 2008). Example questions included: “What were your opinions of the two different screen positions that you saw in the experiment?” and “What physical and emotional sensations were you aware of whilst watching the ball pinches?” Probes were used to ensure a thorough consideration and response from each participant. These included “Can you describe these differences?” and “Was that present during one screen position more than the other, or about the same?”.

Following this, all participants completed a bespoke questionnaire focusing on their affect and experiences during each screen condition. Example questions included: “How strongly did you feel that the hand you were watching was your own?” and “How strong was the feeling that you were performing the movement?” A 6-point Likert scale recorded responses ranging from 0 “*not at all like my own*” to 5 “*strongly like my own*”, and 0 “*no feeling at all*” to 5 “*very strong feeling*” respectively. Each question was answered once for each screen position, to allow for a comparison between the horizontal and vertical screen position. For the question, “On which screen did you prefer watching the ball pinch?”, a single 7-point Likert scale was used to allow for a middle, “*no preference*”, response.

### Data Analysis

#### Overall TMS Data

Electromyographic activity 200 ms prior to the TMS pulse was checked to identify trials with increased muscle activity immediately prior to the stimulation. Trials in which the baseline peak-to-peak amplitude was 2.5 standard deviations greater than the mean baseline were discarded from further analysis (Loporto et al., 2012). This process resulted in 4% of all trials being discarded. To account for inter-individual variability in TMS-induced activity, raw MEP data were transformed into *z*-scores (Fadiga et al., 1995; Loporto et al., 2012) prior to analysis with a 2 (screen position: horizontal, vertical) × 2 (video: action, static) repeated measures ANOVA on all participants’ MEP data.

#### Questionnaire and Interview Data

The lead author used typical descriptors of motor imagery and action observation, such as visual perspective and movement agency (Holmes and Calmels, 2008), to code the interviews and develop the deductive themes. Additionally, the third author independently coded a portion of the interviews using the same descriptors of motor imagery and action observation. The codes generated by the two authors were discussed within the experimental team until agreement was reached to ensure reliable coding and consistent use of terminology. Prior to the deductive thematic analysis, the complete interview coding was discussed at length within the experimental team in order to categorize preference. Following agreement by all members of the experimental team, the data were analyzed using a deductive thematic analysis, following the procedure outlined by Braun and Clarke (2006). Coding and data management were facilitated using NVivo qualitative data analysis software (version 11). Strategies to enhance analytic rigor included comparisons of themes between the questionnaire and interview responses. The themes and questionnaire response comparisons were verified further following discussion with the wider research team to ensure they were comprehensive and inclusive in relation to the themes relating to screen position preference. Paired sample *t*-tests were used on the questionnaire data to compare the responses for the horizontal and vertical screen positions. These were then compared to the interview data where appropriate.

#### Screen Position Preference TMS Data

Based on the analyses of the qualitative data, the MEP data were grouped by screen position preference. A 2 (screen position: horizontal, vertical) × 2 (video: action, static) repeated measures ANOVA was then performed on the horizontal preference (*n* = 16, 5 female, 3 left-handed) group data. Descriptive data only is presented for the vertical (*n* = 7, 2 female, 3 left-handed) group data due to the small number of participants reporting a preference for this screen position. No analysis was performed on the no preference group data (*n* = 1, female, right-handed). Additionally, descriptive data is provided plotting the difference in MEP amplitude between action observation on the horizontal and vertical screens against screen position preference.

## Results

### Overall TMS Data

**Figure 2:**
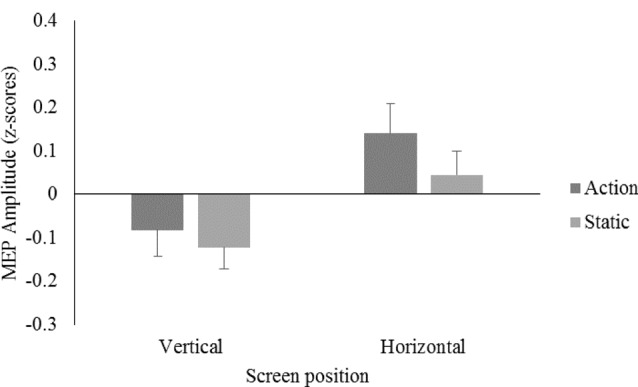
Mean motor evoked potential (MEP) amplitudes displayed as *z*-scores, for the action and static conditions on the vertical and horizontal screen position for the right first dorsal interosseous (FDI) muscle.

A 2 (screen position) × 2 (video) repeated measures ANOVA on data from all participants showed no significant main effects for screen position, *F*_(1,23)_ = 3.11, *p* = 0.09, $\eta_{\text{p}}^{2}$ = 0.12, or video, *F*_(1,23)_ = 1.40, *p* = 0.25, $\eta_{\text{p}}^{2}$ = 0.06, and no significant screen position × video interaction, *F*_(1,23)_ = 0.47, *p* = 0.50, $\eta_{\text{p}}^{2}$ = 0.02 (see Figure 2).

### Questionnaire and Interview Data

The horizontal and vertical screen position conditions and elements related to participants’ viewing experiences of these conditions (e.g., visual perspective, movement agency, movement kinesthesis, peripersonal space) provided the structure for the thematic analysis. Analysis of the interviews suggested a primary theme of self-agency, relating to the participants’ kinesthetic experience and self-agency realism was associated with both screen positions but to a greater or lesser extent depending on screen preference. Data from the interview and questionnaire are presented under the deductive themes of realism and movement ownership, and imagery emerging from the interview.

#### Realism and Movement Ownership

Videos presented on the horizontal screen position were generally perceived to be more “realistic” (e.g., participant (P)6; P18; P21) than those presented on the vertical screen (“Because it doesn’t look like my hand. Especially because my hand was not in the same position” (P24)) and this was associated with perceived ownership of the observed limb: “it [the hand/arm] did seem like mine, more on the [horizontal screen]” (P22). Participants reported that this was, in part, aided by the congruent positioning of their own hand/arm with the model’s hand/arm. Participants reported that the positional congruency enhanced the ownership of the hand/arm (“I felt it was where my hand was, I actually felt like I was looking at my hand” (P8); “I felt like when it was flat [horizontal screen], it was easier to identify as my own [hand]” (P23)), promoted perceived interaction with the video (“it felt like my hand was going into the screen as it was under the screen” (P3)), and provided a greater sense of movement ownership (“When it was on the horizontal screen…… and my hand was underneath the screen, it made me feel like it was my hand that was moving” (P21)).

In support of the qualitative data suggesting screen position differences in affect and preference, *t*-tests on the questionnaire responses to the question “How strongly did you feel that the hand you were watching was your own?” supported the contention that participants experienced a significantly greater ownership of the observed hand in the horizontal screen condition compared to the vertical screen condition *t*_(23)_ = 4.15, *p* < 0.001, $\eta_{\text{p}}^{2}$ = 0.43. An increased sense of embodiment with the observed hand also gave some participants a desire to perform, or a perception that they were actually performing, the observed movement. For example, when observing the videos on the horizontal screen, some participants reported that they “felt as though [he/she] wanted to act that same movement” (P6), or that he/she wanted to actively interact with the movement and “grab it [the ball] when it [the hand] was squeezing it [the ball]” (P4).

#### Imagery

The interviews indicated that some participants employed concurrent imagery behavior whilst observing the action. During the interview, 21 participants reported the spontaneous use of imagery in some form, with nine discussing a range of imagery modalities in detail. These were raised during varying questions, including “What were your opinions of the two different screen positions that you saw in the experiment?” and “What physical and emotional sensations were you aware of whilst watching the ball pinches?” For example, participants reported they felt like they were “doing [the movement] with [their] brain” (P14), and even generated kinesthetic and haptic imagery (“the feeling of the, what seemed to be like a stress ball kind of material” (P18)), and auditory (“I could kind of hear it going, like a noise to it” (P23)) elements to the image. Participants who reported experiencing these multimodal images also reported that they were generated to a greater extent, but not exclusively, during the observation of the ball pinch on the horizontal, compared to the vertical, screen position, and during the action, compared to static, videos. If they did not raise the differences themselves, participants were prompted to compare the different conditions.

Interview data suggested that the horizontal screen position, with its associated imagery, gave participants the perception that they were actively involved in performing the movement. Participants reported feeling “as though [they were] grasping the ball” and that this may have been due to action observation on the horizontal screen generating tactile sensations, such as feeling “… the resistance of the ball in the ball squeezing” (P23). From the questionnaire data, *t*-tests on the responses to the question “How strong was the feeling that you were performing the movement?” confirmed that participants had a greater feeling that they were performing the movement when observing videos on the horizontal screen compared to the vertical screen *t*_(23)_ = 4.63, *p* < 0.001, $\eta_{\text{p}}^{2}$ = 0.48.

### Screen Position Preference Data

**Figure 3:**
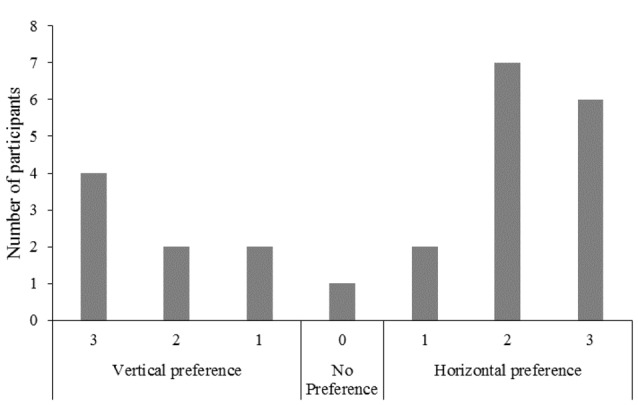
Frequency of responses for the question “On which screen did you prefer watching the ball pinch?”.

In response to the question “On which screen did you prefer watching the ball pinch?”, seven (29%) participants reported a vertical screen position preference, 16 (67%) reported a horizontal screen position preference, and one (4%) indicated no preference for either screen position (Figure 3).

### Screen Position Preference TMS Data

**Figure 4:**
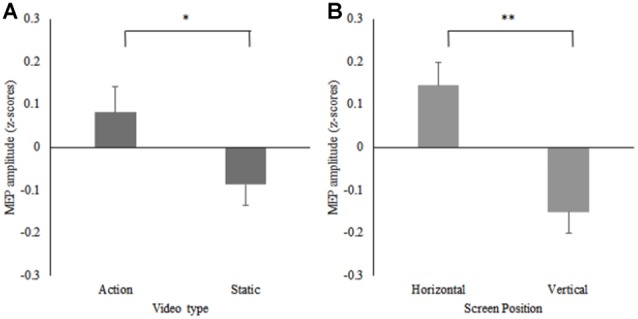
Mean MEP amplitudes displayed as *z*-scores from the horizontal preference group, for **(A)** the action and static conditions (*p = 0.01) and **(B)** the horizontal and vertical screen positions (**p = 0.03) for the right FDI muscle.

**Figure 5:**
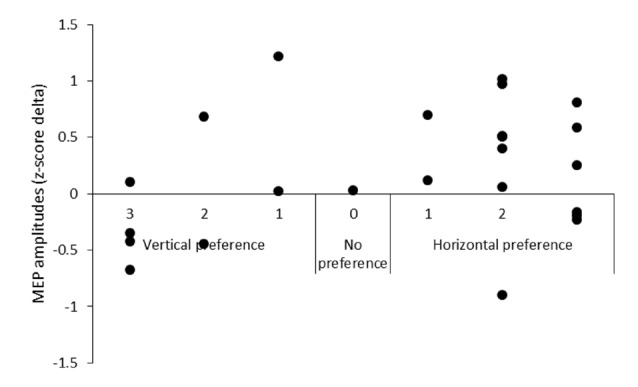
The difference in MEP amplitude between action observation on the horizontal and vertical screen positions plotted against screen position preference.

A 2 (screen position) × 2 (video) repeated measures ANOVA on the data from participants who reported a horizontal screen position preference (*n* = 16) revealed significant main effects for screen position *F*_(1,15)_ = 6.05, *p* = 0.03, $\eta_{\text{p}}^{2}$ = 0.29, and video *F*_(1,15)_ = 8.38, *p* = 0.01, $\eta_{\text{p}}^{2}$ = 0.36. MEPs were significantly greater during observation of the action videos compared to the static videos irrespective of screen position and significantly greater during trials on the horizontal screen position compared to the vertical screen position irrespective of video type. No significant screen position × video interaction effect was found *F*_(1,15)_ = 2.29, *p* = 0.15, $\eta_{\text{p}}^{2}$ = 0.13 (see Figure 4). Due to the small number of participants who reported a preference for the vertical screen position (*n* = 7), only descriptive data is presented for this group (see Table 1). Additionally, descriptive data plotting the difference in individual participants’ MEP amplitudes obtained during action observation on the horizontal and vertical screens is presented against their subjective screen position preference scores for all participants (see Figure 5).

**Table 1:** Mean motor evoked potential (MEP) amplitudes displayed as *z*-scores, for the action and static conditions on the vertical screen position for the right first dorsal interosseous (FDI) muscle.

	Horizontal screen position		Vertical screen position	
	Action	Static	Action	Static
Mean MEP	−0.12 ± 0.10	−0.002 ± 0.12	0.03 ± 0.12	0.10 ± 0.03
amplitude

## Discussion

The first aim of this experiment was to determine whether different screen positions modulate corticospinal excitability during the observation of a hand movement filmed from a first person visual perspective. In contrast to our hypothesis, there were no significant differences in MEP amplitude between the two different screen positions or the type of video presented. The lack of significant difference in MEP amplitude when viewing action compared to static hand videos was surprising as, since Fadiga et al.’s (1995) seminal study, research has generally demonstrated that action observation produces an increased corticospinal excitability in the muscle(s) used to perform the action when compared to a static control (Loporto et al., 2011; Naish et al., 2014). Despite this well-established facilitation effect during action observation, some studies have, however, reported that only certain types of action observation facilitate corticospinal excitability in comparison to control conditions (e.g., Enticott et al., 2010; Donne et al., 2011). Collectively, the results from these studies indicate that facilitation of corticospinal excitability is more likely when the action is perceived as particularly meaningful to the individual. In part, we suggest that the extent to which the action was considered meaningful by each participant varied because of their preference for the screen positions, and may explain the lack of significance. The qualitative data suggested that 67% of individuals preferred viewing the action on the horizontal screen. Participants with the horizontal screen viewing preference, may have attributed less meaning to the videos on the vertical screen due to the different reference frame, thereby introducing a confound to MEP results when screen position viewing preference was not considered. When the TMS data control for screen preference as a marker of attributed meaning, the experimental vs. control effect becomes evident within the participants who indicated a preference for the horizontal screen condition. This will be discussed in more detail later.

A second aim was to establish whether corticospinal excitability was modulated when accounting for participants’ viewing preference for screen position. Follow-up analysis of TMS data for just those participants who preferred the horizontal screen position demonstrated significantly larger MEPs for this sub-group when they observed videos displayed on the horizontal screen compared to the vertical screen. Furthermore, and consistent with the literature (e.g., Loporto et al., 2011; Naish et al., 2014), MEPs were also significantly larger during the observation of the ball pinch action compared to the observation of the static hand. These data suggest that screen position and its induced viewing preference can highlight differences in MEP amplitude and could explain the lack of significance between conditions in the initial analysis.

Seventy-one percent of participants reported that they perceived the observed hand as their own sex. This may have contributed to participants’ sense of ownership and self-agency of the observed hand. Additionally, during the horizontal screen position, anatomical and perceptual congruency with the physical task is emphasized. Combined, this may have resulted in the participants reporting a greater sense of ownership and self-agency during the horizontal screen position compared to the vertical screen position. These qualitative data suggest that the horizontal screen position optimally presented participants with visual and affective cues to reinforce self-attribution for the movement of the action.

The greater corticospinal excitability during the videos presented on the horizontal screen position in participants with a preference for the horizontal screen position suggests that the sense of agency was increased for these participants. We propose that, in part, this may be a function of the dorsal visual stream for these participants during the videos on the horizontal screen. Amongst participants with a horizontal screen position preference, the vertical screen position may require an imagined rotation of the observed image to provide an “ego-relative remapping” (Filimon, 2015) of the hand in an attempt to experience the richer motor simulation only present in their preferred environment. When the reference frame requires the remapping of the action there is predominantly activation of ventral, rather than dorsal, visual stream processing (Filimon, 2015; van Polanen and Davare, 2015). It is possible, therefore, that greater activation of dorsal posterior-parietal pathway is present during the horizontal screen position when the observer holds a preference for it. This may explain some of the contribution to the larger MEPs during the preferred horizontal condition when compared to the inferotemporal pathway of the ventral stream associated with the non-preferred vertical screen condition, although further research is required to test this postulate.

Further mechanistic explanation for the screen position effect on corticospinal excitability can be found in Jeannerod’s (2001) work. He proposed that activation of the motor cortex and descending motor pathways during action observation generates signals that propagate upstream to parietal and premotor cortex which allow monitoring of the simulation and a realization that the participant is the agent of the covert activity, even though there is no overt behavior. Therefore, for the horizontal screen preference group, the greater corticospinal excitability suggests that the screen position generates cortical activity that is associated with a greater feeling of self-identification and, therefore, ownership and self-agency of the observed action, even though they are viewing a model’s arm and hand producing the action. The qualitative data also highlighted that not only did the horizontal screen position give a significantly greater sense of self-ownership, but that it also gave a more realistic kinesthetic feeling about performing the movement compared to the vertical screen position. The viewer can only use visual, imagined kinesthetic, and predicted proprioceptive information to make a judgment about the sense of ownership of the hand and limb in the two conditions. However, in action observation conditions, the latter is significantly compromised, placing greater emphasis on the visual and kineasthetic cues. On the horizontal screen, the congruence of the visual perception and kinesthetic imagery with the predicted proprioceptive information from the viewer’s own arm seemed to have provided greater ownership and agency of the movement in contrast to the vertical screen position where the visual perceptions are incongruent to the observer’s kinesthesis and expected proprioceptive feedback. These findings concur with studies using the rubber hand illusion (Schütz-Bosbach et al., 2009). The importance of vision’s contribution to the sense of ownership and movement agency has been shown extensively in these studies with the authors concluding, in line with our findings, that motor facilitation depends strongly on the agent to whom the observed action is attributed.

In our study, the illusion of a sense of ownership and realism extended to the perception of haptic afference of the ball’s texture and kinesthetic sensations associated with finger flexion but, for most participants, only whilst viewing the action on the horizontal screen during the action condition. This supports similar findings from Farnè et al. (2000) who showed that the brain can form visual representations of a non-owned body part. The authors identified that the rubber hand illusion was only evident when participants saw the rubber hand as congruent to the positioning of their own hand. In contrast, and in support of why corticospinal excitability was significantly lower during the vertical screen in the horizontal preference group, the illusion was significantly reduced when the position of the rubber hand was incongruent to the observer’s own hand. It was suggested that this phenomenon is due to the dominance of vision over proprioception in the perception of limb ownership. More specifically, provided that they look plausible with respect to the subject’s own body, the visual cues of a “fake” arm and hand become self-attributed and a sense of ownership may arise. In the case of our study, the horizontal screen does this whereas the vertical screen presents a misaligned posture that requires imagined rotation to regain positional plausibility.

In a study with some similarities to our own, Kaneko et al. (2007) reported a facilitation of corticospinal excitability and kinesthetic experience when participants observed abduction movements of an index finger from an egocentric viewpoint on a horizontal screen. A vertical screen presentation, however, resulted in reduced kinesthesis and lower corticospinal excitability. Based on our findings and interpretation of the data, the vertical screen position provided participants with a different spatial reference frame and reduced visual cues for self-agency and ownership compared to the horizontal screen. This could be further explained by what Jeannerod and Pacherie (2004) have described as an error in self-predication because the vertical screen presents the viewer’s arm/hand as their own, but in a visually incongruent position to their own that contributes to proprioceptive error. The proprioceptive error can be seen through lower levels of corticospinal excitability in the vertical screen position compared to the horizontal screen position for the horizontal preference group. In order to maintain a sense of limb and movement ownership, a recalibration of the position is required, similar to the ego-relative remapping process described by Filimon (2015). Participants may experience an enhanced kinesthesis during action observation when the feeling of where their hand is meant to be is congruent with where they see the modeled hand. This supports the notion that it is important to ensure action observation tasks that aim to mimic self-actions are delivered from an egocentric reference frame in peripersonal space and filmed from a plausible anatomical viewpoint.

The evidence from the qualitative data suggests that participants used concurrent coordinative imagery during the horizontal screen position, but possibly not during the vertical screen position. In line with the arguments presented above, the differences visual cues between the two screen positions and the associated kinesthesis may be contributory to the difference in MEP amplitude. Vogt et al. (2013) proposed stronger activations in motor execution-related areas when the observed and imagined tasks are fully congruent, as would be proposed here for the horizontal screen condition. In contrast, the lower MEPs for the vertical screen position could be associated with the less congruent observation-imagery behavior, with the observed action only being coordinative with the imagery, which was not reported to have been employed, in order to rotate the image of the hand and retain the perception of self. The horizontal screen condition seems to have given participants the perception that they were performing the observed action and experiencing greater kinesthetic imagery, shown through facilitated corticospinal activity (Stinear et al., 2006). Due to the more congruent visual perspective participants may have found it easier to use appropriate imagery in the horizontal screen position and this, in turn, may have contributed to the greater MEPs.

Taken together, these results demonstrate that anatomical and perceptual congruency with the physical task, alongside the consideration of participants’ screen position viewing preferences, have the potential to modulate corticospinal excitability during action observation. These findings, therefore, have important implications for the design and delivery of action observation interventions in motor (re)learning settings. Specifically, structured action observation interventions have been shown to contribute significantly to improvements in motor function in situations where an individual’s movement capability has been compromised, for example following a stroke or in individual’s with Parkinson’s disease (Buccino, 2014). Despite the apparent efficacy of action observation as an adjunct to physical therapy for motor rehabilitation, these interventions continue to present first person visual perspective action observation on vertically-orientated screens in the observer’s extrapersonal space and screen position viewing preference is rarely considered. Whilst not completely supported by the MEP data, the results within the horizontal screen position preference group alongside the qualitative data indicate that these variables may be important in order to optimize action observation interventions. Importantly, advances in mobile information technology now allows for the relatively easy creation and delivery of action observation interventions via tablet and smartphone devices (McCormick and Holmes, 2016). The portability of such devices makes it considerably easier to manipulate the positioning of the screen, where appropriate for the task being viewed, to achieve perceptual and anatomical congruency with the observed action, and match the individual’s screen position viewing preference. As such, future research should seek to expand on these TMS findings, and establish the efficacy of integrating screen position preference into action observation interventions for motor (re)learning within clinical populations.

## Author Contributions

MR, DJW, ZCF and PSH all contributed to the design of the experiment. MR was responsible for participant recruitment, data collection and data analysis. MR, DJW, ZCF and PSH all contributed to the writing of the manuscript.

## Conflict of Interest Statement

The authors declare that the research was conducted in the absence of any commercial or financial relationships that could be construed as a potential conflict of interest.
